# Therapeutic potential of astrocyte transdifferentiated neurons

**DOI:** 10.4103/NRR.NRR-D-25-00554

**Published:** 2025-12-30

**Authors:** Xiaojun Liang, Rongxing Qin, Qingchun Qin, Wei Xu, Hongyu Xu, Xinyu Lai, Lingduo Shao, Caiqi Li, Minshan Xie, Xiaoyuan Xiong, Qi Tang, Li Chen

**Affiliations:** 1 Department of Neurology, The First Affiliated Hospital of Guangxi Medical University, Nanning, Guangxi Zhuang Autonomous Region, China; 2 National Center for International Research of Biological Targeting Diagnosis and Therapy (Guangxi Key Laboratory of Biological Targeting Diagnosis and Therapy Research), Guangxi Medical University, Nanning, Guangxi Zhuang Autonomous Region, China

**Keywords:** Alzheimer’s disease, amyotrophic lateral sclerosis, cell transdifferentiation, Huntington’s disease, ischemic stroke, nervous system diseases, Parkinson’s disease, ptbp1 protein, small molecule, spinal cord injury, transcription factors

## Abstract

The permanent functional deficits resulting from the inability of adult mammalian central nervous system neurons to regenerate after injury present a significant clinical challenge. While traditional stem cell transplantation strategies continue to encounter ethical concerns and the risk of immune rejection, this impasse has shifted regenerative medicine research toward targeting endogenous astrocytes. Due to their intrinsic plasticity, widespread distribution throughout the central nervous system, and affinity for neurodevelopmental lineage, astrocytes are a unique target for *in situ* neuronal regeneration. This review systematically elucidates the core regulatory network governing astrocyte transdifferentiation, identifying 10 key signaling pathways, such as Wnt signaling pathway, that form a cascade regulatory system. Directed overexpression of transcription factors such as NeuroD1, Ascl1, or Neurog2 can directly initiate neuronal phenotypic conversion. Meanwhile, small molecule compounds such as valproic acid combined with CHIR99021 activate endogenous neurogenic programs by inhibiting the bone morphogenetic protein signaling axis. Notably, polypyrimidine tract binding protein 1 (*PTB*) gene silencing significantly enhances transdifferentiation efficiency by suppressing the microRNA 124/re1 silencing transcription factor (miR-124/REST) feedback loop. From a translational perspective, a multidimensional evaluation system based on morphological, molecular marker, and electrophysiological properties has demonstrated considerable therapeutic potential. In stroke models, NeuroD1-mediated transdifferentiation replenished approximately 30% of lost cortical neurons and improved motor coordination, evidenced by enhanced performance in food pellet retrieval, grid walking, and cylinder tests compared with controls. In spinal cord injury studies, SOX2-induced glutamatergic neurons moderately reduced glial scar density by about 25%, permitting regenerating axons to pass through while preserving the supportive structure of scar. In neurodegenerative contexts, *PTB* inhibition yielded functionally mature dopaminergic neurons and reconstructed nigrostriatal pathways in Parkinson’s disease models. In Alzheimer’s disease models, adeno-associated virus-delivered NeuroD1 induced whole-brain neural circuit remodeling, generating 500,000 new neurons widely distributed across the cortex and hippocampus, accompanied by improved cognitive performance. Current technical limitations include off-target effects of adeno-associated virus vectors, which cause nonspecific gene expression and require rigorous validation via Cre-loxP lineage tracing. Transdifferentiation efficiency is also highly influenced by regional microenvironments: gray matter astrocytes show higher conversion rates than those in white matter, and oxidative stress increases apoptosis among newly generated neurons. Clinical translation is further constrained by the safety of delivery systems and the aging tissue microenvironment, where transforming growth factor beta 1 is often elevated. Ferroptosis inhibitors have been shown to nearly double the survival rate of transdifferentiated cells, offering a novel strategy to mitigate oxidative damage. Based on current evidence, astrocyte transdifferentiation enables neural functional recovery across multiple disease models through endogenous repair mechanisms. Future advances should focus on optogenetically inducible vectors for spatiotemporal precision, non-viral delivery systems to mitigate vector-related risks, and integration of long-term safety validation in non-human primates with single-cell multi-omics technologies to facilitate the clinical translation of personalized regenerative therapies.

**Facts**

• Research has shown that astrocytes can be efficiently converted into functional neurons through transcription factor–mediated transdifferentiation techniques, with conversion efficiencies exceeding 80% in mouse models. These neurons can integrate into local neural circuits, leading to improved behavioral outcomes.

• A combination of small molecule compounds has successfully induced the conversion of astrocytes into neurons by activating the Wnt/GSK-3β signaling pathway, eliminating the need for genetic manipulation and providing new possibilities for clinical applications.

• Inhibition of the RNA-binding protein Ptbp1 has been shown to directly convert astrocytes into neurons, offering a novel strategy for neural repair that does not require gene editing.

**Open questions**

• How do the epigenetic barriers and chronic inflammation in the aging human brain or complex pathological microenvironments limit the efficiency of transdifferentiation of astrocytes?

• What long-term evaluation models and monitoring strategies are necessary to assess the synaptic stability, electrical activity safety, and potential tumorigenic/seizure risks of transdifferentiated neurons over their decades-long lifespan?

• How can we design a non-viral delivery system that features cell subtype–specific targeting, efficient penetration of the blood–brain barrier, and precise spatiotemporal release, in order to achieve clinically affordable personalized neuroregenerative therapies?

## Introduction

Impaired neural regeneration after central nervous system (CNS) injury is a fundamental pathological mechanism behind functional deficits in stroke, spinal cord injury (SCI), and neurodegenerative disorders. Embryonic neurons exhibit robust regenerative potential; however, adult mammalian neurons have intrinsically limited regenerative capacity, constrained by inhibitory microenvironmental cues and cell-autonomous properties (Li et al., 2025a, b; Tang et al., 2025; Wang et al., 2025). Traditional stem cell transplantation holds therapeutic promise but faces significant challenges, including ethical controversies, immune rejection, and barriers to functional integration (Talifu et al., 2024; Geng et al., 2025). In recent years, endogenous astrocytes have emerged as a novel focus in regenerative medicine due to their ubiquitous distribution, phenotypic plasticity, and proximity to neurogenic lineages (Gerber and Perrin, 2024).

Astrocytes, the most abundant glial cell population in the CNS (20%–40%), adopt a reactive phenotype under pathological conditions, forming glial scars that sequester lesions (Li et al., 2021). These cells can be reprogrammed through transdifferentiation into functional neurons. This strategy directly harnesses proliferative cells within injury sites as an *in situ* cellular reservoir, offering a transformative approach for neural repair.

**Figure 1:**
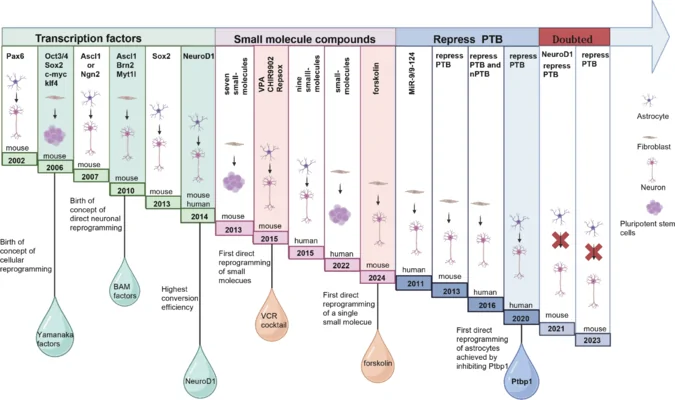
Timeline of key milestones in astrocyte transdifferentiation into neurons. Created with BioRender.com. Ascl1: Achaete-scute family BHLH transcription factor 1; Brn2: POU class 3 homeobox 2 (POU3F2); c-myc: MYC proto-oncogene, BHLH transcription factor (MYC); Klf4: KLF transcription factor 4; Myt1l: myelin transcription factor 1 like; NeuroD1: neuronal differentiation 1; Ngn2: neurogenin 2; nPTB: polypyrimidine tract binding protein 2 (PTBP2); Oct3/4: POU class 5 homeobox 1 (POU5F1); Pax6: paired box 6; PTB: polypyrimidine tract binding protein 1 (PTBP1); Sox2: sry-box transcription factor 2; VPA: valproic acid.

Transcription factor–mediated transdifferentiation achieves over 80% conversion efficiency in murine models, yielding neurons that integrate into local circuits and improve behavioral outcomes (Chen et al., 2020). Small molecule cocktails bypass genetic manipulation by activating endogenous neurogenic programs through modulation of Wnt/GSK-3β signaling (Yang et al., 2020). Inhibition of the RNA-binding protein polypyrimidine tract binding protein 1 (Ptbp1) induces direct astrocyte-to-neuron conversion (**Figure 1**).

The field faces substantive critiques. Studies reporting “converted neurons” have been challenged for potential misidentification of endogenous neurons due to viral vector off-target effects or inadequate lineage tracing (Wang et al., 2021). Glial scars were traditionally viewed as primary barriers to regeneration; however, emerging evidence indicates their dual role in containing damage, limiting inflammatory spread, and providing structural support (Huang et al., 2024). Complete scar ablation strategies may incur unforeseen consequences. Numerous experimental approaches have successfully induced axonal regeneration or long-distance growth in animal models, yet these nascent axons frequently fail to establish functional synaptic connections with target neurons, resulting in limited functional recovery. Most studies rely on rodent models, which exhibit significant differences from humans in injury mechanisms, regenerative capacity, and neural circuit complexity (Tai et al., 2021; Lentini et al., 2021). Additional translational barriers include the low targeting specificity of current viral vectors, the limited CNS penetration efficiency of non-viral delivery systems (e.g., nanoparticles), and the unverified long-term safety of transdifferentiated neurons. These knowledge gaps critically impede clinical translation and demand systematic evaluation.

Addressing these challenges, this review aims to systematically elucidate the molecular mechanisms and technological advances in astrocyte transdifferentiation, emphasizing the influence of the pathological microenvironment on transdifferentiation efficacy. It will critically analyze cellular identity controversies, with an emphasis on rigorous lineage tracing methodologies, and integrate therapeutic efficacy data from preclinical disease models (e.g., stroke, Parkinson’s disease) to establish cross-species validation frameworks and optimize delivery strategies. This review introduces the concept of “spatiotemporally specific transdifferentiation,” advocating combinatorial approaches using nanocarriers and immunomodulatory technologies to overcome microenvironmental constraints. Distinct from previous reviews, this work integrates critical controversy analysis, microenvironmental interactions, and clinical translation roadmaps within a unified framework, providing theoretically grounded and clinically actionable guidance for endogenous repair–based neural regeneration strategies.

To avoid ambiguity, this review defines the following key concepts: Transdifferentiation refers to the process by which a differentiated somatic cell directly transitions into another distinct lineage-specific, typically differentiated cell type under specific inductive conditions, without reverting through a pluripotent stem cell state. Conversely, reprogramming primarily denotes induced pluripotent stem cell (iPSC) reprogramming in this work, which involves reverting differentiated somatic cells to a pluripotent state via defined factors; however, the term broadly encompasses transdifferentiation. Conversion (or direct reprogramming), largely synonymous with transdifferentiation, describes the directed *in vitro* induction of one cell type into another target phenotype using genetic or chemical factors, bypassing pluripotency. Notably, “transdifferentiation” is specifically used throughout this manuscript to describe the direct transformation of astrocytes into neurons observed in our experimental system.

## Search Strategy

**Figure 2:**
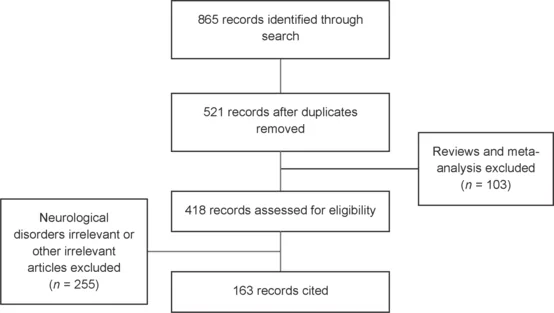
Flowchart of data retrieval and inclusion and exclusion strategies. Created with BioRender.com.

A literature search was conducted across three biomedical databases (PubMed, Medline, and Google Scholar), encompassing records from their inception through July 2025, with particular emphasis on seminal studies and recent high-impact publications (2020–2025). The Boolean search query ((transdifferentiation) OR (reprogramming)) AND ((astrocyte) OR (astroglial cell)) was deployed, followed by title and abstract screening. Inclusion criteria required original research on astrocyte-to-neuron conversion published in English, while exclusion criteria eliminated non-English publications, studies of exogenous stem cell transplantation without transdifferentiation components, and non-peer-reviewed sources, including conference abstracts and patents. The two-stage screening process involved an initial assessment of 865 records based on titles and abstracts, followed by a full-text eligibility evaluation of 418 articles; content discrepancies were resolved through a tripartite consensus. Ultimately, 163 publications meeting all inclusion criteria were retained for analysis (**Figure 2**).

## Biological Properties and Functions of Astrocytes

Astrocytes are the most widely distributed and functionally complex subtype of glial cells in the CNS, accounting for 20%–40% of the total number of glial cells in the human brain (Li et al., 2021). They serve not only as metabolic supporters of neurons but also as central regulators of neural development, synaptic plasticity, and damage repair (Iida, 1968; Spitzer et al., 2013).

In terms of physiological functions, astrocytes are involved in the construction and dynamic regulation of the blood–brain barrier (BBB), the provision of metabolic support to neurons, the maintenance of ionic homeostasis, and the regulation of synaptic plasticity and neurotransmitter levels (Tang et al., 2023). Astrocyte end-feet cover about 99% of the cerebral vascular surface and promote the formation of tight junctions between endothelial cells through the secretion of laminin and fibronectin, providing structural support to the BBB (Teleanu et al., 2018). Inwardly rectifying potassium channels (Kir4.1) in the cell membrane of astrocytes actively take up excess extracellular K^+^ to prevent neuronal overexcitation (Ohno et al., 2021). Astrocytes dynamically modulate synaptic function through a “tripartite synapse” mechanism, releasing D-serine as a co-agonist of N-methyl-D-aspartate (NMDA) receptors to enhance long-term potentiation (LTP), while adenosine triphosphate modulates synaptic strength by activating neuronal P2X receptors (Lenk et al., 2019).

Astrocytes perform a range of critical functions throughout neural development, actively participating in both the formation of synapses (synaptogenesis) and the subsequent maturation of these synapses, as well as playing a key role in the regulation of myelination (Rodrigues et al., 2019). These cells actively promote synapse formation through the secretion of specific proteins, including thrombospondin (TSP1/2) and cholesterol carrier proteins such as apolipoprotein E (ApoE), which facilitate the establishment of structural connections between presynaptic and postsynaptic membranes (Rudenko, 2017).

Astrocytes play a complex role in CNS inflammation with dual regulatory properties (Alsbrook et al., 2023; Bai et al., 2024). On one side, astrocytes perform protective functions by forming glial scars through the upregulation of glial fibrillary acidic protein (GFAP) (Bai et al., 2024) and wave proteins, which isolate injury sites and limit peripheral immune cell infiltration. On the other side, astrocytes drive harmful processes by promoting neurodegeneration and sustaining neuroinflammation through several pathogenic mechanisms. They release harmful substances such as reactive oxygen species and excitatory amino acids, such as glutamate, which exacerbate neuronal oxidative damage. Additionally, over-activated STAT3 signaling in astrocytes promotes the deposition of scarring-associated molecules, with chondroitin sulfate proteoglycans as key examples, and these chondroitin sulfate proteoglycans inhibit axonal regeneration (Alsbrook et al., 2023).

Astrocytes possess a remarkable proliferative reserve and a close genealogical affinity with neurons. These properties enable them to achieve higher transdifferentiation efficiency and cell survival rates while also promoting integration into peripheral neural circuits for long-term functionality. Collectively, these multiple advantages establish astrocytes as the optimal starting cells for neuronal transdifferentiation.

## Core Mechanisms Underlying Astrocyte-to-Neuron Transdifferentiation

The transdifferentiation of astrocytes to neurons is synergistically regulated by multiple key molecular pathways, including the Wnt–GSK-3 pathway. Among these, gene editing technology-mediated overexpression of transcription factors, combinatorial interventions with specific small molecule compounds, and gene silencing strategies targeting PTB proteins represent the current core strategies.

### Key signaling pathways and transcriptional regulatory networks

#### Wnt-GSK-3 signaling pathway

**Figure 3:**
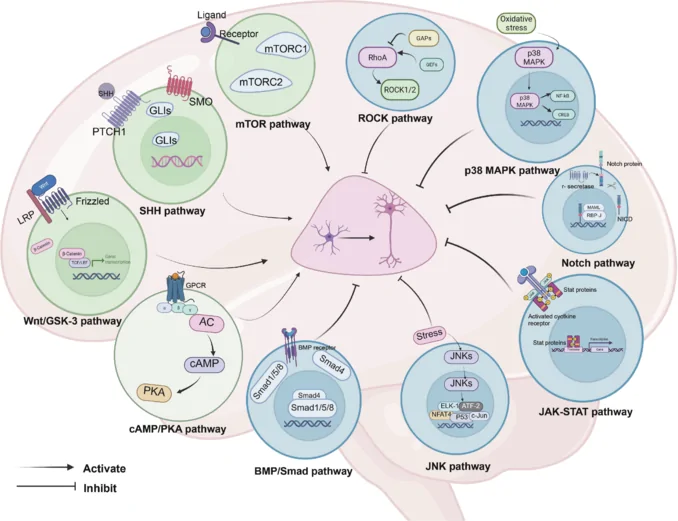
Signaling pathways of neuron transdifferentiation. Created with BioRender.com. AC: Adenylyl cyclase; ATF-2: activating transcription factor 2; BMP: bone morphogenetic protein; cAMP: cyclic adenosine monophosphate; c-Jun: Jun proto-oncogene, AP-1 transcription factor subunit; CREB: cyclic AMP-responsive element-binding protein; ELK-1: ETS transcription factor ELK1; GAPs: GTPase-activating proteins; GEFs: guanine nucleotide exchange factors; GLIs: the zinc finger transcription factors; GPCR: G-protein coupled receptor; JAK-STAT: Janus kinase-signal transducer and activator of transcription; JNK: Jun N-terminal Kinase; LEF1: lymphoid enhancer factor 1; LRP: receptor-related protein; MAML: mastermind-like protein; mTOR: mammalian target of rapamycin; mTORC1: mTOR complex 1; mTORC2: mTOR complex 2; NFAT4: nuclear factor of activated T cells 3; NF-κB: nuclear factor kappa-light-chain-enhancer of activated B cells; NICD: Notch intracellular domain; p38 MAPK: P38 mitogen-activated protein kinase; PKA: protein kinase A system; PTCH1: receptor patched 1; RBP-J: recombining binding protein suppressor of hairless; RhoA: Ras homologous family member A; ROCK: Rho-associated protein kinase; SHH: sonic hedgehog; SMO: receptor smoothened; TCF: T-cell factor; Wnt/GSK-3: (Wingless/integrated)/glycogen synthase kinase 3.

Wnt proteins are a highly conserved family of secreted glycoproteins (Pan et al., 2022). Notably, Frizzled receptors, which operate independently of low-density lipoprotein receptor-related proteins 5/6 (LRP5/6), function as Wnt ligand-binding entities. They activate β-catenin-independent signaling cascades through alternative effector mechanisms (Wei et al., 2012; Seitz et al., 2014). This results in altered cell motility and polarity, as well as antagonism of the β-catenin pathway (**Figure 3**).

Activation of Wnt signaling has been reported to promote the transdifferentiation process, while inhibition of GSK-3 signaling facilitates homeostatic maintenance and the induced generation of neural progenitor cells (Li et al., 2020; Pu et al., 2023). Pharmacological inhibition of GSK3β using selective antagonists (CHIR99021/kenpaullone) induces the indirect activation of canonical Wnt-β-catenin signaling in murine and human pluripotent systems. Additionally, the synergistic application of CHIR99021 with epigenetic modulators enhances astroglial-to-neuronal transdifferentiation efficiency through the coordinated regulation of lineage-specific transcription factors (Liang et al., 2024).

#### Sonic hedgehog signaling pathway

Sonic hedgehog (Shh) acts as a pleiotropic morphogen critical for embryonic central nervous system morphogenesis and organ patterning (Hung et al., 2016). Signaling is initiated when Shh binds to its receptor Patched (Ptc), a process that relieves the inhibition of Smoothened (Smo). The activation of Smo then triggers intracellular signaling, which ultimately activates Gli transcription factors (Arveseth et al., 2021). A critical balance exists between Gli activators and repressors at specific locations and times, allowing neural tissue to interpret Shh concentration gradients. These morphogen gradients are decoded precisely, enabling primitive neuroepithelia to function by transforming gradient information into distinct neuronal subtype identities (Cohen et al., 2015; **Figure 3**).

After mechanical or ischemic brain injury, astrocytes increase Shh production, which enhances their ability to form neurospheres in laboratory cultures—neurospheres being indicators of stem-like potential. Shh also acts as a patterning signal, with cells interpreting Shh concentration gradients to direct top-bottom patterning in the developing neural tube. This is achieved by controlling transcription factors such as Olig2 and Nkx6.1, processes that determine the development of ventral motor neurons (Jessell, 2000). Activating Shh shows therapeutic promise: using the drug SAG (a smoothened agonist) triggers the direct conversion of human fetal astrocytes into functional neurons under laboratory conditions, and these new neurons can generate electrical signals (Zhang et al., 2013).

#### Mammalian target of rapamycin signaling pathway

The mammalian target of rapamycin (mTOR) is a serine/threonine kinase that consists of two complexes: mTOR complex 1 (mTORC1) and mTOR complex 2 (mTORC2), and is regulated by other proteins (Panwar et al., 2023; **Figure 3**).

mTOR signaling affects cell survival during brain injury, impacting neuronal viability in cerebral ischemia. Research shows that suppressing mTOR activity can protect neurons, as inhibiting mTOR safeguards dopaminergic neurons by promoting the autophagic clearance of α-synuclein deposits (Ravikumar et al., 2004). Pharmacological activation of mTOR shows promise: using the drug valproic acid (VPA) boosts mTOR signaling, which enhances cellular transdifferentiation efficiency and promotes neuronal differentiation (Duan et al., 2019).

#### Cyclic adenosine monophosphate/protein kinase A signaling pathway

The cyclic adenosine monophosphate (cAMP)/protein kinase A (PKA) signaling cascade functions as a key downstream effector of G-protein coupled receptors, transducing physiological responses to multiple hormonal and neurotransmitter signals. As the primary mammalian cAMP receptor, PKA exerts its biological effects through the phosphorylation of diverse protein substrates, including ion channels (**Figure 3**), metabolic enzymes, and gene regulatory factors, thereby modulating their functional states. Within cells, cAMP biosynthesis occurs via the adenylate cyclase (AC)-mediated conversion of adenosine triphosphate, a process initiated by stimulatory Gα subunits (Gαs) following Gs-coupled receptor activation (De Jong et al., 2025).

Research has established that small molecules targeting the cAMP/PKA pathway can effectively overcome the influence of the *in vivo* environment on cellular transdifferentiation (Xu et al., 2019). Specifically, forskolin (a cAMP/PKA activator) promotes the conversion of fibroblasts into functional neurons (Hu et al., 2019; Cardon et al., 2020; Wang et al., 2024a, b). Additionally, forskolin enhance intracellular cAMP levels, producing neurotrophic effects while reducing oxidative stress damage and improving cell survival (Xu et al., 2019).

#### The JAK–STAT signaling pathway

The JAK–STAT pathway is a major signaling system that helps cells respond to cytokines. This pathway is initiated when a ligand, such as hormones, growth factors, or cytokines, binds to a receptor, activating JAK kinases, which then phosphorylate themselves. This process triggers STAT protein activation (**Figure 3**). Activated STAT proteins form dimers (pairs) that move into the cell nucleus, where they bind to specific DNA sequences to regulate gene transcription (Song et al., 2017; Wang et al., 2022a, b).

STAT3 activation plays a beneficial role in cell transdifferentiation, working with the factors Oct4, Sox2, and Klf4 to enhance the efficiency of generating iPSCs (Zhang et al., 2019b). Inhibiting the JAK–STAT pathway can facilitate neuronal conversion, as demonstrated by the inhibitor ruxolitinib, which improves the efficiency with which rat cortical astrocytes transform into neurons (Zhang et al., 2019a, b). Another JAK2 inhibitor, AZ960, works differently by promoting mesenchymal-to-epithelial transition through the indirect inhibition of STAT3 signaling, an action that enhances the conversion of fibroblasts into neurons (Wang et al., 2022a, b).

#### Bone morphogenetic protein-Smad signaling pathway

Bone morphogenetic proteins (BMPs) belong to the transforming growth factor-beta (TGF-β) superfamily and act as pleiotropic extracellular morphogens (Wu et al., 2024). The SMAD pathway is the main signaling system within cells, functioning just downstream of activated BMP receptors. This pathway transduces BMP signals into cellular responses through specific steps. First, activated type I receptors phosphorylate receptor-activated SMADs (R-SMADs), which include SMAD1, SMAD5, SMAD8, and SMAD9. Second, phosphorylated R-SMADs bind to SMAD4, a co-mediator of SMAD, forming a complex that moves into the nucleus, where it regulates gene transcription (Goodnough et al., 2012; Jiang et al., 2025). Experimental evidence confirms the importance of SMAD. Knocking out SMAD4 in specific brain cells impairs neurogenesis in stem/glial populations in the subventricular zone. Blocking BMPs with Noggin also disrupts this process, demonstrating that SMAD signaling regulates adult brain plasticity (Gámez et al., 2013). Pharmacologically targeting this axis influences cell fate; inhibiting both TGF-β and BMP4 together steers iPSCs toward neural ectoderm, promoting subsequent neuronal differentiation (Bataille et al., 2020). Additionally, BMP inhibition protects brain tissue. Overexpressing Noggin in injury models preserves myelin integrity following hypoxic-ischemic damage. Notably, dual drug inhibition enables direct conversion: using Dorsomorphin (targeting BMP) alongside SB431542 (targeting TGF-β) successfully transforms human peripheral T lymphocytes into functional neurons (Tanabe et al., 2018). Moreover, the BMP inhibitor LDN193189 promotes the transdifferentiation of human astrocytes into neurons (Ma et al., 2019; Yin et al., 2019).

#### Notch signaling pathway

The Notch signaling cascade exerts pivotal regulatory roles in cellular activation dynamics, mitotic control, lineage specification, and programmed cell death (Sprizak and Blacklow, 2021; Martin et al., 2023). Distinct from conventional signaling paradigms, Notch receptors require triphasic proteolytic processing before the nuclear translocation of their intracellular domains (NICD) for direct transcriptional modulation (**Figure 3**).

Constitutive Notch signaling in mature astrocytes maintains their non-neurogenic state, with pharmacological inhibition (e.g., LY411575) suppressing GFAP promoter methylation while enhancing neuronal differentiation potential (Ohuchi et al., 2019; Zhang et al., 2022). The proneural transcription factor ASCL1, governed by Notch-induced repressors HES1 and HES5, orchestrates neurogenic programs through the spatiotemporal regulation of neural stem cell proliferation and differentiation. Post-transcriptional regulation via miR-153 modulates Notch pathway activity through the coordinated targeting of the JAG1 ligand and HES/HEY effectors, establishing an inverse relationship between neurogenic competence and gliogenic potential (Sun et al., 2023).

#### p38 mitogen-activated protein kinase signaling pathway

Mitogen-activated protein kinases (MAPKs) are serine/threonine kinases that show regulated expression in both neuronal and non-neuronal cells within the mature CNS (Corrêa and Eales, 2012). These enzymes respond to diverse signals. The MAPK family has several key subgroups, including c-Jun N-terminal kinases 1–3 (JNK1–3) and p38 isoforms (α, β, γ, and δ). The JNK and p38 subgroups are stress-activated kinases that respond strongly during disease states (Troy et al., 2001; Zhu et al., 2005). All MAPK isoforms need dual phosphorylation for activation. Specific threonine and tyrosine residues must be phosphorylated together (**Figure 3**).

Combined treatment using SB203580 (a selective p38α/β inhibitor) and the neural-specific miR-124 triggers the conversion of cortical reactive astrocytes into functional neurons in rodent models, underscoring the critical role of isoform-specific mechanisms in regulating neural plasticity (Zheng et al., 2021).

#### Ras homologous family member A–Rho-associated kinases signaling pathway

Ras homologous family member A (RhoA) is a key signaling protein, with particularly critical roles in neurodevelopment by controlling neuronal migration, differentiation, and proliferation (Liu et al., 2023). The primary effectors of RhoA signaling are Rho-associated kinases (ROCK) I and II, serine/threonine kinases regulated by the small GTPase RhoA (**Figure 3**; Zhang et al., 2023). This cycling process is precisely controlled by three regulatory protein families: guanine nucleotide exchange factors, GTPase-activating proteins, and guanine nucleotide-dissociation inhibitors.

Experimental studies demonstrate that inhibiting the Rho/ROCK pathway enhances neurite growth in spinal cord injury models. ROCK inhibitors have been shown to promote both neuronal differentiation and neurite extension (Boomkamp et al., 2012; Giraldo et al., 2021). The ROCK inhibitor Y-27632 specifically blocks Rho activity, a major GTPase effector. In mice with corticospinal tract injuries, treatment with Y-27632 improved both functional and anatomical recovery. Additionally, when used as part of a chemical cocktail, Y-27632 can convert human cells into neurons (Wan et al., 2018).

#### c-Jun N-terminal kinase signaling pathway

c-Jun N-terminal kinases (JNKs) constitute a major branch of the MAPK superfamily. The JNK activation cascade relies on sequential phosphorylation, mediated by upstream MAPK kinases, specifically MKK4 and MKK7 (**Figure 3**; Caliz et al., 2021). Members of the MAP3K family regulate these upstream kinases. Following activation via phosphorylation, JNKs translocate into the nucleus, where they regulate key transcription factors, including activating transcription factor 2 and the jun proto-oncogene (c-Jun), as well as nuclear hormone receptors (Kirsch et al., 2020). Importantly, JNK functionality extends beyond nuclear regulation, as JNKs also phosphorylate diverse cellular substrates.

Substantial evidence demonstrates that oxidative stress accompanies cellular transdifferentiation, and JNK pathway suppression significantly improves the viability of induced neuronal (iN) cells. In the context of transdifferentiation, the JNK-specific inhibitor SP600125 collaborates with other small molecules to drive efficient conversion of somatic cells into neurons (Hu et al., 2015, 2019; Liu et al., 2020a).

### Multidimensional targeting strategies for astrocyte-to-neuron transdifferentiation

#### Overexpression of transcription factors

Transcription factors exert precise genomic control through sequence-specific recognition of DNA motifs, modulating transcriptional activation and repression to establish spatiotemporally restricted gene expression patterns. These molecular mediators possess distinct structural motifs that enable high-affinity binding to target-specific genomic loci, thereby governing transcriptional dynamics through the regulation of chromatin accessibility.

The cellular transdifferentiation paradigm underwent a significant shift following Takahashi and Yamanaka’s seminal demonstration that murine fibroblasts (from both embryonic and somatic origins) could acquire pluripotency through the combinatorial delivery of a quartet of transcription factors (Oct3/4, Sox2, c-Myc, and Klf4) (Takahashi and Yamanaka, 2006). This breakthrough established the molecular blueprint for iPSC generation, catalyzing exponential growth in research on transdifferentiation mechanisms. Subsequently, several studies have successfully transdifferentiated various types of cells into iPSCs, including umbilical cord blood cells (Giorgetti et al., 2009), T lymphocytes (Brown et al., 2010), and astrocytes (Li et al., 2017). The therapeutic potential of iPSCs spans three-dimensional biomedicine, including regenerative interventions, pathophysiological modeling, and pharmacological screening. Particularly noteworthy is the transdifferentiation competency of astrocytes, which demonstrate superior efficiency in cellular transdifferentiation compared to other somatic cell lineages. Multiple transcription factors, as well as single transcription factors (Zfp521, DLX2, and SOX2), have been shown to reprogram astrocytes into inducible neural progenitors (Ruiz et al., 2010; Corti et al., 2012; Niu et al., 2013; Zarei-Kheirabadi et al., 2019; Zhang et al., 2022). Recently, it has been shown that neural progenitor cells generated from DLX2-induced astrocytes can differentiate into neurons, astrocytes, and oligodendrocytes in mice, facilitating the repair of dysfunctions in all three neural lineages caused by nerve injury. However, classical malignancy features, such as inactivation of the p53 pathway or silencing of the inhibitor of kinase 4/alternative reading frame locus, have been reported in both somatic cell transdifferentiation and cell transformation. Additionally, human iPSCs are more prone to developing into teratomas than human embryonic stem cells. The risk of teratoma formation increases when transdifferentiation involves an intermediate state that includes iPSCs.

After continuous attempts, subsequent studies overcame this challenge. Pioneering work by Heins et al. (2002) revealed that neural progenitor cells with radial morphology in cortical regions possess neuron-generating capacity, with the transcriptional regulator Pax6 functioning as a key epigenetic determinant governing gliogenic-to-neurogenic lineage commitment. Overexpression of Pax6 using retroviruses enables cortical astrocytes cultured *in vitro* to produce microtubulin beta-III and NeuN-positive neurons (Heins et al., 2002). However, this finding of astrocyte transformation into neurons did not attract enough attention from the academic community. It was not until 2006 that Yamanaka’s discovery led more researchers to invest in this area. Again, because iPSCs carry a certain risk of forming teratomas, people began to seek ways to achieve transdifferentiation.

The molecular cocktail Ascl1/Brn2/Myt1l (BAM) demonstrates potent neurogenic transdifferentiation efficacy in somatic fibroblasts, enabling murine embryonic and postnatal-derived cell populations to acquire mature neuronal phenotypes exhibiting electrophysiological competence and synaptogenic capacity under defined culture conditions (Caiazzo et al., 2015). Meanwhile, topology can enhance the efficiency of BAM transdifferentiation of fibroblasts (Mattiassi et al., 2021). Subsequently, BAM directly reprogrammed terminally differentiated hepatocytes into neurons, demonstrating that transdifferentiation can be achieved between cell types of different embryonic origins (Marro et al., 2011). Subsequent mechanistic studies have demonstrated that Ascl1 is a pioneer factor in the BAM cocktail. Ascl1 can rapidly occupy many homologous genomic sites in fibroblasts at an early stage and can simultaneously recruit Brn2 to its site to play a role. The study also identified a key target gene of Ascl1, Zfp238, which can partially replace Ascl1 during transdifferentiation (Wapinski et al., 2013). The monogenic delivery of Ascl1 suffices to reprogram diverse somatic cell lineages (murine/human fibroblasts) and embryonic stem cells into electrophysiologically active induced neuronal cells, validating the identity of Ascl1 as a key transdifferentiation driver. At the same time, MYT1L and BRN2 play a role in promoting neuronal maturation (Chanda et al., 2014). Overexpression of Ascl1 and histone deacetylase inhibitors in adult mice with retinal injury converts Müller glial cells into neurons (Jorstad et al., 2017). Beyond fibroblast transdifferentiation, Ascl1 facilitates astrocyte-to-neuron transdifferentiation, generating electrophysiologically active iNs. However, the iNs mature more slowly than embryonically born neurons (Berninger et al., 2007). Early binding of Ascl1 to neuron-enriched mitochondrial antioxidant proteins (especially PRDX2 and SOD1) accelerates the transformation process of astrocytes and prolongs the lifespan of induced neurons, resulting in more mature neuronal morphology and longer axons (Russo et al., 2021).

After Ascl1, overexpression of the transcription factor NeuroD1 alone was found to induce transdifferentiation of astrocytes into functional neurons. Additionally, NeuroD1 was effective in transdifferentiating human astrocytes cultured *in vitro* into functional neurons (Guo et al., 2014). Subsequently, AAV9, which possesses intrinsic blood-brain barrier penetrance, was delivered to mouse astrocytes via the intravascular route to overexpress NeuroD1. Ninety-nine percent of the astrocytes in the vascular system were specifically infected and were able to transform into neurons (Brulet et al., 2017). However, astrocytes at different sites exhibit varying transdifferentiation abilities. White matter astrocytes demonstrate marked resistance to NeuroD1-induced conversion, resulting in neurons that resemble their white matter-derived counterparts (Liu et al., 2020b).

Furthermore, the proneuronal transcription factor neurogenin-2 (NEUROG2) possesses a distinct capacity to directly reprogram cortical astrocytes into glutamatergic neurons, as evidenced by relevant research findings (Berninger et al., 2007; Heinrich et al., 2010). *In vivo* studies focused on astrocyte-to-neuron conversion have revealed that targeted overexpression of NEUROG2 in astrocytes located in the murine midbrain and spinal cord can induce transdifferentiation; the neurons generated through this process exhibit mature electrophysiological properties and successfully achieve region-specific integration into neural circuits, confirming the efficacy of *in vivo* transdifferentiation. Neurons primarily depend on oxidative phosphorylation for energy production, while astrocytes rely on anaerobic glycolysis and fatty acid β-oxidation to meet their energy needs. During the process of astrocytic conversion, the demands of metabolic transdifferentiation, along with the consequent high-level oxidative stress, create significant barriers that hinder neuronal transdifferentiation. Research outcomes indicate that ferroptosis inhibitors and antioxidants play a significant role in enhancing the efficiency of direct astrocyte-to-neuron conversion. Supplementing Bcl-2 in transdifferentiation protocols involving NEUROG2 or Ascl1 not only substantially improves the yield of neurons but also generates functional neurons with electrophysiological activity (Gascón et al., 2016). Additionally, the combinatorial delivery of NEUROG2 and the nuclear receptor Nurr1 enables region-specific astroglial transdifferentiation with an efficiency exceeding 80% in spiked mice; the induced neurons in these cases establish laminar-specific molecular markers and extend axonal projections over long distances (**Additional Table 1**). It is critical to note that NEUROG2 requires Nurr1 as an obligate molecular partner to achieve high-fidelity conversion, and this synergistic interaction appears to be mediated through the dual capacity of Nurr1 to suppress neuroinflammatory pathways and mitigate the oxidative stress associated with transdifferentiation (Mattugini et al., 2019; Liu et al., 2021).

**Additional Table 1:** Representative transcription factors involved in *in vitro* and *in vivo* direct astrocyte-to-neuron conversion and transdifferentiation studies

Transcription factor	*in vitro/in vivo*	Cell type/animal model	Vector/delivery system	Induced neuronal phenotype	Functional outcome/electrophysiology	Induction efficiency	Reference
Pax6	*In vitro*	Astrocyte (mouse)	Retrovirus	β-Tubulin-III, NeuN, GABA or glutamate transporter-1 positive neurons	Not described	50%	Heins et al., 2002
Ascii	*In vitro*	Astrocyte (mouse)	Retrovirus	β-Tubulin-III	Receive synaptic input from cocultured neurons; Hodgkin-Huxley-type action potentials, i.e., voltage-dependent Na^+^ and K^+^ channels	37%	Berninger et al., 2007
Ascii + Bcl2	*In vitro*	Astrocyte (mouse)	Plasmid	DCX, NeuN, and MAP2	Action potentials consistent with fully developed neurons	90%	Gascón et al., 2016
Ascii (add mitochondrial antioxidant proteins, such as Prdx2 and Sodi)	*In vitro*	Astrocyte (mouse)	Retrovirus; dCas9 VPR coding plasmid; Rosa26-loxP-Stop-LoxP-dCas9VPR-SAM mice	β-Tubulin-III	Not described	10%-15%	Russo et al., 2021
NeuroD1	*In vivo*	Astrocyte (mouse)	Retrovirus	DCX, NeuN, Tbr1, Ctip2	Large sodium currents and potassium currents; robust spontaneous synaptic events; survive for at least 2 months in mouse brain	92.80%	Guo et al., 2014
	*In vitro*	Astrocyte (mouse)	Retrovirus	NeuN, VGluT1, Ctip2, Otx1, Tbr 1	Large GABA, glutamate, and NMDA currents; repetitive action potentials and large Na^+^ and K^+^ currents	> 90%	
	*In vitro*	Astrocyte (human)	Retrovirus	DCX, NeuN, and MAP2, VGluT1, Ctip2, Otx1, Tbr1, SV2	Same as above	90%	
NeuroD1	*In vivo*	Astrocyte (mouse)	AAV9; intravascular route	NeuN	Not described	> 10%	Brulet et al., 2017
	*In vitro*	Astrocyte (mouse)	Lentivirus	β-Tubulin-III	Not described	Not described	
Ngn2	*In vitro*	Astrocyte (mouse)	Retrovirus	β-Tubulin-III, Tbr1	Receive synaptic input from cocultured neurons; Hodgkin-Huxley-type action potentials, i.e., voltage-dependent Na^+^ and K^+^ channels	71%	Berninger et al., 2007
Ngn2	*In vitro*	Astrocyte (mouse)	Retrovirus/plasmid	β-tubulin-III, map2, vGluT1, Tbr2, Map2	Repetitive action potentials	70%	Heinrich et al., 2010
Ngn2 + Bcl2	*In vivo*	Astrocyte (mouse)	Retrovirus	DCX, NeuN, Ctip2, FoxP2	Not described	75%	Gascó n et al., 2016
Ngn2 + Nurr1	*In vivo*	Astrocyte (mouse)	mGFAP Cre mice, FLEx switch (Cre-On) AAV	NeuN, CUX1, CTI P2^+^	Develop morphological and functional synaptic connections and long-distance axonal projections; action potentials consistent with fully developed neurons	53%	Mattugini et al., 2019

Ascl1: Achaete-scute family BHLH transcription factor 1; Bcl2: BCL2 apoptosis regulator; Ctip2: BCL11 transcription factor B (BCL11B); DCX: doublecortin; FoxP2: forkhead Box P2; GABA: γ-aminobutyric acid; MAP2/map2: microtubule associated protein 2; NeuN: RNA binding fox-1 Homolog 3 (RBFOX3); NMDA: N-methyl-D-aspartate; Otx1: orthodenticle homeobox 1; Pax6: paired Box 6; Prdx2: peroxiredoxin 2; Sod1: superoxide dismutase 1; Tbr1: T-box brain transcription factor 1; vGluT1: solute carrier family 17 member 7 (SLC17A7).

#### Incorporation of small chemical molecules

Small molecule compound transdifferentiation is a method of cell fate transdifferentiation that does not involve genetic manipulation, unlike the use of transcription factors or microRNAs (Yang et al., 2020). Small molecules orchestrate genome-wide transcriptional networks through the modulation of signaling cascades and chromatin state remodeling. However, because they are not integrated into the genome, this method offers a relatively flexible and controllable approach to transdifferentiation (Yang et al., 2020).

Yamanaka’s team achieved a breakthrough in reprogramming (Takahashi and Yamanaka, 2006). However, four of Yamanaka’s factors are oncogenes, such as c-Myc and Klf4, prompting researchers to search for chemicals that could replace these oncogenes. Through continuous exploration, these four factors were gradually replaced by small chemical molecules, ultimately achieving reprogramming using only small molecules—a significant breakthrough with strong application potential.

In 2013, a major achievement emerged when Hongkui Deng’s team induced chemical pluripotency in murine somatic lineages through the iterative optimization of a defined seven-molecule cocktail (Hou et al., 2013). The resultant iPSCs exhibited embryonic stem cell-equivalent pluripotency across molecular and functional hierarchies. For the first time, it was demonstrated that an exogenously provided “master gene” program is not necessary for pluripotent somatic cell reprogramming, showing that cell fate reprogramming can successfully occur using only small molecules to initiate an endogenous pluripotency program (Hou et al., 2013). However, due to the stability and lower plasticity of the human cell epigenome, reprogramming of human cells into human pluripotent stem cells via chemical methods was not realized until 2022. This study emphasizes the importance of an intermediate plasticity state and identifies the JNK pathway as a major obstacle to chemical transdifferentiation. Inhibition of the JNK pathway enhances cellular plasticity and suppresses the inflammatory response, thereby facilitating the transdifferentiation of human fibroblasts (Guan et al., 2022).

Chemical transdifferentiation involves multiple steps and offers greater flexibility, allowing the direction and efficiency of induction to be altered by simply adjusting the composition and concentration of small molecule combinations. Experimental investigations demonstrated that murine astroglial populations undergo transdifferentiation into neurons via a cocktail of VPA, CHIR99021, and Repsox, whereas their human counterparts exhibit recalcitrance to such chemical transdifferentiation paradigms. A cocktail of small-molecule compounds was able to directly reprogram human embryonic astrocytes into functional neurons within 8–10 days. This combination included nine small molecules, and it was found that the transdifferentiation process involves the activation of endogenous transcription factors NGN1/2, NEUROD1, and Ascl1. Most of the neurons transformed in this study were glutamatergic neurons that exhibited robust spontaneous synaptic events and survived for months in culture. However, this protocol may only be suitable for human brain-derived astrocytes, as human spinal cord astrocytes cannot be effectively directly reprogrammed (Zhang et al., 2015). Following the successful transformation of human embryonic astrocytes, human adult astrocytes were also reported to be transformed into functional neurons using a combination of small molecules: VPA, CHIR99021, Repsox, forskolin, i-Bet151, and ISX-9. Critically, intracerebroventricular engraftment of the induced neuronal derivatives in neonatal murine hosts resulted in sustained survival and progressive acquisition of electrophysiological functionality (Gao et al., 2017). Given that the complex manipulation of too many small molecule combinations poses challenges for clinical application, researchers sought to explore chemical transdifferentiation methods with fewer small molecule components. Building on this, the researchers found that a quadruplet pharmacological formulation enables the transdifferentiation of human fetal astroglial populations into excitatory neuronal phenotypes, surpassing the efficacy of the nine-molecule combination in transdifferentiation efficiency. These four molecules can be replaced by small molecules targeting the same signaling pathways (Notch, GSK-3 β, TGF-β, and BMP), highlighting the dominance of these pathways (Ma et al., 2019).

Ma et al. (2021) used an osmotic mini-pumping system to inject a small molecule cocktail into mature murine CNS, achieving the transdifferentiation of resident astroglial cells into functional neuronal populations. This experiment involved injecting adeno-associated virus (AAV-FLEX-EGFP) into the brains of transgenic Aldh1l1-cre mice to label astrocytes. They combined this with specific retrograde neuronal tracing to rule out the possibility of incorrect reorganization of the Cre, thereby double-checking the correct origin of the induced neurons. Recent research demonstrates the monopharmaceutical transdifferentiation of human skin fibroblasts into electrophysiologically active neurons via Forskolin-mediated cAMP activation. The neurons exhibited sustained survival exceeding 60 days across both *ex vivo* and *in vivo* environments (Wang et al., 2024a, b). The realization of transdifferentiation by a single small chemical molecule represents a significant advance in the field. However, whether this approach can be applied to other cell types remains to be explored.

#### Inhibition of the neurogenic negative regulator polypyrimidine tract binding protein 1

**Figure 4:**
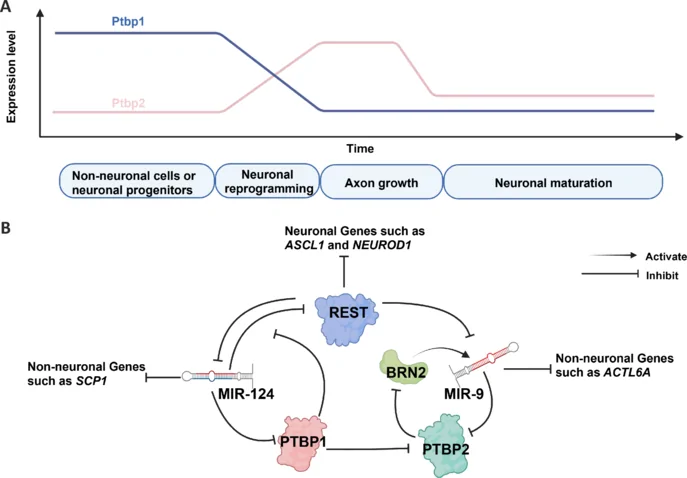
Dynamic regulation of Ptbp1 and Ptbp2 during neurogenesis. (A) Early in neurogenesis, Ptbp1 levels progressively decline, while Ptbp2 expression is transiently induced. (B) The two molecular circuitries of PTBP1 and PTBP2. The REST complex suppresses numerous neuron-specific genes, including Ascl1 and NEUROD1. Conversely, miR-124 and miR-9 repress multiple non-neuron-specific genes. Upon PTBP1 knockdown, miR-124-mediated suppression of the REST complex is enhanced, leading to disassembly of multiple components within the REST complex. Consequently, increased miR-124 expression redirects cellular fate toward the neuronal lineage. In the PTBP2 circuitry, PTBP2 downregulation elevates BRN2 expression, which subsequently induces miR-9 upregulation, thereby promoting the differentiation of neuron-like cells into functional neurons. Created with BioRender.com. ACTL6A: Actin like 6A; ASCL1: achaete-scute family BHLH transcription factor 1; BRN2: POU class 3 Homeobox 2 (POU3F2); MIR: microRNAs; NEUROD1: neuronal differentiation 1; Ptbp1: polypyrimidine tract binding protein 1; Ptbp2: polypyrimidine tract binding protein 2; REST: RE1 silencing transcription factor; SCP1: synaptonemal complex protein 1.

PTB family members (polypyrimidine tract binding protein 1 [Ptbp1] and polypyrimidine tract binding protein 2 [Ptbp2]) function as dual ribonucleoprotein regulators, orchestrating alternative splicing dynamics through sequence-specific RNA interactions (Makeyev et al., 2007; Zheng et al., 2012). Variable splicing denotes the generation of distinct exons within a single pre-mRNA transcript through different splicing methods. This splicing can result in multiple similar but different mRNAs for the same gene at different developmental stages or differentiation/physiological states, yielding proteoforms harboring shared core domains yet exhibiting divergent functional profiles (Li et al., 2014). Ptbp1 (PTB) is widely expressed in non-neuronal and neuronal progenitor cells (NPC) but is repressed in differentiated neurons. In contrast, Ptbp2 (also known as nPTB) is present at low levels in NPC. However, when NPC was induced to differentiate into postmitotic neurons, nPTB was dramatically upregulated, promoting axonogenesis (Boutz et al., 2007). During neurodevelopment, PTB and nPTB exhibit reciprocally regulated expression dynamics; genetic ablation of PTB triggers compensatory nPTB upregulation (**Figure 4**). The switch in expression of these two allows the splicing of many exons to be reprogrammed, promoting neuronal differentiation and maturation (Zhang et al., 2019a, b).

PTB has splicing regulatory capacity and affects microRNA function by competitively binding to target mRNAs or altering local RNA secondary structures (Boutz et al., 2007; Makeyev et al., 2007). Neuron-specific microRNAs (miR-9/9* and miR-124) serve as pivotal regulators of neurogenic transdifferentiation, with miR-124 ectopic expression enhancing embryonic stem cell neural lineage specification (Maiorano and Mallamaci, 2009). Yoo et al. (2011) demonstrated that miR-9/9* combined with miR-124 (miR-9/9*-124) induces neuronal transdifferentiation in human fibroblasts, albeit with a suboptimal yield (< 5% efficacy). However, the conversion efficiency was greatly improved when combined with the neurogenic transcription factor NEUROD2. Given the close relationship between PTB and microRNAs, researchers have conducted in-depth studies on the function of PTB in transdifferentiation (Xue et al., 2013). It was subsequently shown that inhibition of PTB expression is sufficient to drive the transdifferentiation of murine embryonic fibroblasts into neuronal phenotypes. This lineage conversion operates via the PTB-REST-miR-124 loop. MiR-124 regulates the REST-constructed transcriptional silencing complex, which epigenetically silences neuronal genes (including miR-124 itself) in non-neuronal cells. Crucially, miR-124 and neural microRNAs inhibit the REST complex (SCP1/CoREST), thereby forming an autoregulatory loop. PTB inhibition initiates a cascade activation of this miR-124/REST switchpoint, triggering REST complex destabilization and promoting lineage commitment toward a neuronal identity.

Inhibiting PTB can transform murine embryonic fibroblasts into electrophysiologically competent neurons, whereas activation of the PTB-REST-miR-124 loop in human fibroblasts yields phenotypically immature neuronal derivatives (**Figure 4**; Xue et al., 2016). Only by sequentially knocking down PTB and nPTB could human fibroblasts be successfully transformed into mature neurons, suggesting that there are differences between human and mouse cells. This sequential process involves another key self-enhancing loop (nPTB-BRN2-miR-9/9*). Within this regulatory loop, nPTB suppression initiates BRN2 signaling activation, which subsequently upregulates miR-9 expression. The functional engagement of this molecular cascade drives the maturation of neuronal progenitor cells into electrophysiologically active neurons. However, when this sequential knockdown optimization protocol was applied to mouse embryonic fibroblasts, the cells died rapidly, indicating that mammalian cells have different built-in timing mechanisms (Xue et al., 2016). Weinberg et al. (2017) knocked down PTB in oligodendrocytes in the rat striatum, successfully transforming oligodendrocytes into functional neurons 6 weeks after AAV4 transduction. Three months after AAV4 transduction, 0.04 mm fluorescent microspheres were injected into the pallidum or substantia nigra, and the fluorescent microspheres were detectable in the striatum 2 weeks later, suggesting that the presynaptic endings functioned in retrograde axonal transport (Powell et al., 2016; Weinberg et al., 2017).

Subsequently, Qian et al. (2020) found that the knockdown of PTB alone could convert human and mouse astrocytes into functional neurons, without the need for the sequential knockdown protocol used for transdifferentiating human fibroblasts. Astrocytes and fibroblasts have similar PTB regulatory loops, and miR-124 expression is low in both cell types. However, unlike fibroblasts, astrocytic lineages exhibit marked enrichment of miR-9 and BRN2 expression, indicating that the nPTB regulatory loop in astrocytes is activated. Astrocytes demonstrate unique transdifferentiation competency through monogenic suppression of PTB, achieving direct conversion into functional neuronal lineages (Qian et al., 2020). Utilizing lentiviral vectors encoding Ptbp1-targeting shRNA (shPTB), this investigation achieved efficient transduction in murine and human astroglial populations under controlled culture conditions. Remarkably, 50%–80% of shPTB-transduced cells exhibited characteristic neuronal morphologies along with concurrent expression of neuronal biomarkers (NeuN/NSE). Furthermore, successful conversion of astrocytes into TH-positive neurons was achieved after the injection of AAV2:CMV-loxP-shPTB-RFP (AAV-shPTB) in Gfap-cre transgenic mice in the midbrain. Similar positive results were obtained in a murine model of Parkinson’s disease. Meanwhile, transient inhibition of PTB using antisense oligonucleotides (ASO)-mediated intranuclear RNase H1 had effects similar to those of shPTB, with both approaches transforming astrocytes into neurons (Qian et al., 2020).

## Therapeutic Implications of Astrocyte Transdifferentiation in Neurological Disorders and Injuries

Based on breakthroughs in the field of neuroregeneration and cellular transdifferentiation, a large number of studies have focused on the treatment of ischemic stroke, spinal cord injury, and neurodegenerative disorders (e.g., Parkinson’s disease [PD], Alzheimer’s disease [AD], Huntington’s disease [HD], and amyotrophic lateral sclerosis [ALS]) through transdifferentiation of astrocytes to functional neurons. Several studies have achieved the transdifferentiation of astrocytes into electrophysiologically active neurons in both *in vitro* and animal models, crossing lineage barriers (Chen et al., 2020; Tan et al., 2022; Wu et al., 2025).

### Ischemic stroke

Ischemic stroke is the second leading cause of death worldwide and a major cause of long-term disability, for which there is no cure (Martin et al., 2024). The sudden interruption of cerebral blood flow leads to the rapid death of neurons and other cells within the ischemic core. The massive death of neurons causes irreversible damage to the body after a stroke (Gong et al., 2023). Currently, the clinical treatment strategy is based on early reperfusion through intravenous thrombolysis or mechanical thrombolysis, supplemented by supportive therapy and management of acute complications. However, the limited therapeutic window and potentially serious side effects restrict the use of intravenous thrombolysis and mechanical thrombolysis. *In vivo*, astrocyte-to-neuron conversion within brain lesions restores functional neural networks, constituting a novel cell replacement strategy for CNS repair.

Post-stroke reactive astrocytes exhibit neurogenic potential through indirect lineage conversion. Investigations demonstrate that stroke-induced doublecortin (DCX)^+^ neuroblasts emerge in the lesioned striatum, originating from locally transformed astrocytes rather than from subventricular zone migration. Mechanistically, stroke-induced attenuation of the Notch1 pathway in astrocytes is a prerequisite for neurogenesis, as pharmacological Notch inhibition triggers neurogenic transdifferentiation in striatal and cortical astrocytes (Magnusson et al., 2014). Additionally, Duan et al. (2013) observed GFP-labeled striatal astrocytes transdifferentiating into βIII-tubulin^+^/MAP-2^+^ immature neurons after middle cerebral artery occlusion, maturing into cholinergic, GABAergic, and dopaminergic subtypes with action potential generation and synaptic integration capacity within 14 days (Grande et al., 2013).

In an adult mouse model of ischemic injury, retrovirus-mediated NEUROG2 was able to directly reprogram non-neuronal cells into neurons, although the transdifferentiation efficiency was low. However, it could be slightly improved by the combined application of growth factors, and cells in the striatum were more likely to be transformed relative to those in the cortex (Grande et al., 2013). Chen et al. (2020) established that NeuroD1 mediates the direct lineage conversion of resident murine glia into synaptically competent neurons within their native parenchymal microenvironment. Experimental validation in murine cerebral ischemia models demonstrated that AAV-mediated NeuroD1 delivery to cortical infarct zones induces progressive transdifferentiation of transduced astrocytes into electrophysiologically functional neuronal populations. Notably, the newly regenerated neuronal populations exhibited dual therapeutic efficacy, compensating for 33% of ischemic insult-induced neuronal loss while providing neuroprotection to an equivalent proportion of compromised neural cells, thereby mitigating secondary degeneration. Concurrently, comprehensive behavioral assays in murine stroke models demonstrated a marked enhancement of locomotor and cognitive capacities following NeuroD1-targeted genetic intervention, indicative of robust neurofunctional restoration (Chen et al., 2020). A subsequent primate study established that NeuroD1-mediated ectopic expression in post-ischemic cortical astrocytes achieved 90% lineage conversion efficacy, yielding phenotypically stable neuronal populations that demonstrated over 12 months of survival with substantial synaptic integration capacity, confirming high-efficiency cellular transdifferentiation outcomes. NeuroD1-mediated cellular transdifferentiation also concurrently attenuated neuroinflammatory responses by suppressing the activation of innate immune effector populations, including microglia and macrophages, while promoting tissue microenvironment homeostasis (Ge et al., 2020). The optimized neuroinflammatory milieu facilitated enhanced viability of ischemia-compromised serotonin (5-HT)^+^/GABAergic interneuron subpopulations through selective neuroprotective modulation of inflammatory mediators. Remarkably, this intervention exhibited an extended temporal therapeutic window and sustained cellular transdifferentiation efficacy, indicating the translational potential of NeuroD1-driven astrocyte-to-neuron conversion strategies for stroke treatment across expanded intervention phases (Puls et al., 2020). NeuroD1-mediated lineage-converted neurons exhibit functional maturation and synaptic integration within visual cortical circuits, driving post-ischemic visual recovery through neural network reconstitution (Tang et al., 2021).

### Spinal cord injury

Spinal cord injury constitutes a devastating trauma to the central nervous system, compromising locomotor and somatosensory capacities through structural integrity disruption in affected individuals. The pathological hallmarks of spinal cord trauma encompass sustained neuroinflammatory cascades, progressive axonal disintegration, neuronal depletion, and astroglial scar formation (Clifford et al., 2023; Wang et al., 2024a, b). Notably, astrogliotic scar deposition at lesion sites establishes a regenerative-inhibitory milieu through dense astrocyte-derived extracellular matrix accumulation (McGraw et al., 2001). However, the potential of methylprednisolone to increase complications makes its application very controversial. Astrocyte transdifferentiation in spinal cord injury aims to compensate for neuronal depletion via lineage-converted neural populations, with *in situ* cellular transdifferentiation feasibility experimentally validated in SCI models through synaptic integration with host neural circuitry.

Pioneering research established that SOX2 monotherapy drives astrocyte-to-neural progenitor cell transdifferentiation, generating proliferative induced adult neuroblasts (iANBs) in both mature and senescent murine systems. Pharmacological co-delivery of brain-derived neurotrophic factor/noggin or epigenetic modulation via HDAC inhibitors significantly enhances iANB maturation into synaptically integrated neurons that exhibit electrophysiological competence within endogenous neural circuits (Niu et al., 2013). Subsequent validation by Su et al. (2014) in spinal cord injury models confirmed SOX2-mediated conversion of resident astroglial populations into DCX^+^ neural progenitors, which underwent *in vivo* differentiation into functionally active neurons. Complementary pharmacological intervention using the HDAC inhibitor VPA further promoted neuronal maturation through targeted epigenetic chromatin remodeling. Expanding this paradigm, Wang et al. (2016) implemented AAV vector-mediated Sox2 delivery specifically targeting astroglial scar lesions in contusive spinal cord injury models. This innovative approach achieved dual therapeutic outcomes: 1) Astrocyte-to-neuron transdifferentiation compensated for neuronal loss; and 2) Concurrent attenuation of fibrotic scar formation through selective extracellular matrix remodeling preserved lesion site integrity. The combined effects elicited significant neuroregenerative outcomes via enhanced axonal regrowth. Mechanistically, the p53 signaling axis critically regulates SOX2-dependent glial transdifferentiation dynamics in adult spinal microenvironments: p53 activation accelerates cell cycle exit in SOX2-generated iANBs, whereas targeted p53 suppression expands neural progenitor pools to potentiate neuroblast production (Wang et al., 2016). Crucially, SOX2-mediated *in vivo* astrocyte transdifferentiation necessitates transit through an actively proliferating intermediate progenitor stage, a characteristic that distinguishes this indirect conversion mechanism from direct transdifferentiation methodologies (Niu et al., 2015).

Emerging evidence documents endogenous astroglial populations in murine spinal cords undergoing lineage conversion into neuronal phenotypes via transcriptional regulator modulation. Complementarily, NeuroD1 overexpression redirects astrocytes toward neural stem cell fates and mature neurons in a rat model of spinal cord injury (Chen et al., 2017). Puls et al. (2020) demonstrated that using AAV NeuroD1 gene therapy, reactive astrocytes from spinal cord-injured mice were efficiently converted into mature neurons (> 95%). Most of these induced neurons were glutamatergic and expressed the spinal cord-specific marker Tlx3, allowing them to integrate into local spinal cord circuits (Puls et al., 2020). In another study, researchers employed AAV-mediated Neurog2 delivery in spinal cord microenvironments, achieving astrocyte-to-neuron transdifferentiation (Liu et al., 2021). The resulting induced neurons displayed electrophysiological responsiveness to multisynaptic inputs originating from dorsal root ganglion afferents. Additionally, CRISPRa-mediated transcriptional activation of the endogenous Ngn2/Isl1 loci drives astrocyte-to-motor neuron transdifferentiation in murine spinal cord models. The lineage-converted neurons acquire characteristic cholinergic morphologies and exhibit functional electrophysiological properties indicative of mature motor neuronal phenotypes (Zhou et al., 2021).

The Notch signaling cascade critically governs astroglial fate determination. Pathway suppression is both necessary and sufficient to drive the lineage conversion of adult parenchymal glia into functional neurons within murine cortical networks (Magnusson et al., 2014). Spatiotemporal analysis reveals constitutive Notch1 signaling in adult murine spinal astroglial populations, with pronounced pathway upregulation following contusive spinal cord injury. Genetic attenuation of Notch1 signaling autonomously initiates the lineage conversion of reactive astroglia into neurons within lesioned spinal parenchyma. Significantly, pharmacological inhibition of Notch1 via DAPT monotherapy recapitulated this astrocyte-to-neuron conversion cascade in post-traumatic spinal microenvironments (Tan et al., 2022).

Recent work by Tan et al. demonstrated that systemic administration of a bimolecular regimen (LDN193189/CHIR99021) in murine spinal cord injury models successfully redirected spinal reactive astrocytes toward neuronal lineages. The converted cells exhibited canonical neuronal cytoarchitecture, with functional maturation and sustained survival exceeding 12 months in spinal parenchyma. Notably, this chemical combination comprises established core components of *in vitro* epigenetic transdifferentiation platforms, as evidenced by prior mechanistic studies (Li et al., 2015; Hu et al., 2015; Zhang et al., 2015). LDN193189 functions as a selective pharmacological antagonist targeting BMP receptor-mediated signaling cascades (Gross et al., 1996), while CHIR99021 acts as a WNT agonist (Chambers et al., 2009). Chemical transdifferentiation protocols induce coordinated activation of endogenous neurogenic transcription factors (Ngn2, NeuroD1/2, Myt1l), with the most pronounced transcriptional induction observed for Ngn2 and NeuroD1 (Tan et al., 2024).

### Parkinson’s disease

PD is a progressive neurodegenerative disorder clinically characterized by the selective degeneration of midbrain substantia nigra dopaminergic neurons, leading to cardinal motor symptoms such as resting tremor, bradykinesia, and muscular rigidity. Contemporary therapeutic strategies focus on palliative management through dopaminergic augmentation but fail to halt the underlying neurodegenerative trajectory (McGregor and Nelson, 2019).

Rivetti di Val Cervo et al. (2017) achieved dopaminergic transdifferentiation of human astrocytes through the combinatorial delivery of the transcription factors NeuroD1, Ascl1, and Lmx1a, along with miR218 (NeAL218). Epigenetic modulation via chromatin remodeling and pharmacological activation of the TGFβ, Shh, and Wnt pathways synergistically enhanced conversion efficiency to 16%. In Parkinsonian murine models, NeAL218 alone induced striatal astrocyte-derived induced dopaminergic neurons that exhibited action potential generation and concomitant restoration of motor coordination (Rivetti di Val Cervo et al., 2017).

*PTB* inhibition represents a pivotal advancement in regenerative neurobiology, enabling the conversion of astroglial lineages into neurons. Lentiviral delivery of shPTB successfully induced neuronal transdifferentiation in murine astroglial populations, yielding electrophysiologically functional neurons. AAV-mediated *in vivo* transduction of shPTB further demonstrated region-specific neuronal subtype specification from astrocytes across distinct CNS compartments. In Parkinsonian murine models, AAV-mediated PTB knockdown enabled the transdifferentiation of midbrain astrocytes into functional dopaminergic neurons. These induced neurons reestablished nigrostriatal dopaminergic circuitry, resulting in elevated striatal dopamine concentrations and improved locomotor capabilities (Qian et al., 2020). In a parallel study, Zhou et al. (2020) used CRISPR-CasRx-mediated PTB knockdown via intraocular delivery of AAV-GFAP-CasRx-Ptbp1 in Ai9 (Rosa-CAG-LSL-tdTomato) transgenic mice. Combined with AAV-GFAP-GFP-Cre lineage tracing, this approach revealed tdTomato+ Müller glia-derived cells co-expressing RGC-specific markers (Brn3a/Rbpms) at 4 weeks post-injection, demonstrating CasRx-driven glia-to-retinal ganglion cell conversion in adult retinal tissue. Striatal application of this system in parkinsonian mice similarly converted astrocytes into tyrosine hydroxylase-positive dopaminergic neurons, rescuing motor deficits through nigrostriatal pathway reconstruction.

Giehrl-Schwab et al. (2022) recently engineered a knock-in murine model integrating a bipartite dCas9 transcriptional activation platform (dCAM) coupled with an AAV-delivered intein-mediated split-dCas9 reconstitution system (AAV-dCAS). This strategy enabled CRISPR activation (CRISPRa) of endogenous transcriptional modules, including (Ascl1/Lmx1a/Nr4a2, ALN) and (Ascl1/Lmx1a/NeuroD1/miR-218, ALNe218), through precise epigenomic remodeling. In 6-hydroxydopamine lesioned murine PD models, both methodologies achieved lineage conversion of striatal astrocytes into induced GABAergic neurons (iGABAergicNs). These neurons exhibited functional synaptic integration into striatal microcircuitry, ameliorating aberrant locomotor patterns through inhibitory network modulation. This platform enables multiplexed activation of endogenous transcriptional programs, overcoming the constraint of exogenous transgene dependency inherent in conventional transdifferentiation paradigms.

### Alzheimer’s disease

AD is a progressive neurodegenerative disorder characterized by extensive neuronal depletion in the cortex and hippocampus, with pathological hallmarks including amyloid-β plaque deposition and neurofibrillary tangles containing hyperphosphorylated tau proteins that trigger neuroinflammatory cascades and oxidative damage, ultimately leading to synaptic disintegration and the collapse of neuronal networks. Current therapeutic strategies mainly target early-stage AD pathophysiology, while curative approaches for advanced disease remain elusive, largely due to the failure to achieve neuronal replenishment and restore disrupted neural circuitry through endogenous neuroregenerative mechanisms.

In murine models of AD, cortical astroglial populations were transdifferentiated into electrophysiologically active glutamatergic neurons through retroviral vector-mediated NeuroD1 expression. These lineage-converted neurons exhibited both spontaneous and evoked postsynaptic currents, demonstrating functional synaptic integration into endogenous cortical microcircuitry (Guo et al., 2014). Intracranial delivery of transdifferentiation vectors enables localized neuronal regeneration but remains therapeutically inadequate for AD due to pan-cerebral neurodegeneration affecting neocortical and hippocampal networks. Building upon previous efforts, Wu et al. (2025) engineered a systemic gene therapy platform using BBB-penetrant AAV-PHP.eB vectors encoding NeuroD1, where intravenous administration achieved astrocyte-to-neuron transdifferentiation across multiple brain regions in 5xFAD mice with extensive neuronal depletion. The induced neurons demonstrated proper laminar positioning and established long-range axonal connectivity via anterograde tracing. Most importantly, behavioral tests showed that whole-brain treatment improved cognitive function in AD mice.

Beyond NeuroD1, the miR-302/367 cluster exhibits astrocyte-to-neuron transdifferentiation capacity in AD pathology. Ghasemi-Kasman et al. (2018) established an intracerebroventricular streptozotocin (ICV-STZ)-induced AD murine model, delivering miR-302/367-expressing lentiviral vectors with GFP reporters into the dentate gyrus, complemented by systemic valproate administration. After a 6-month intervention, whole-cell patch-clamp recordings demonstrated that the induced neurons exhibited repetitive action potential firing akin to native neurons. Behavioral assessments revealed enhanced spontaneous alternation rates and improved Morris water maze performance, indicating restored hippocampal-dependent spatial memory consolidation.

### Huntington’s disease

HD is a monogenic neurodegenerative disorder characterized by a triad of motor dysfunction, cognitive decline, and psychiatric disturbances. It is marked by progressive neuronal atrophy and circuit dysfunction within striatocortical networks. Tetrabenazine has been used to treat chorea in HD and other movement disorders, but it has significant side effects (Claassen et al., 2018).

Wu et al. (2020) demonstrated striatal astrocyte-to-GABAergic neuron transdifferentiation in HD murine models (R6/2 and YAC128) through AAV vector-driven ectopic co-expression of NeuroD1/Dlx2, achieving an 80% lineage conversion efficiency. Immunohistochemical analysis confirmed that over 50% of the induced neurons exhibited DARPP32^+^ medium spiny neuron phenotypes characteristic of striatal GABAergic circuitry. The induced neurons displayed mature electrophysiological properties and established functional connectivity with the globus pallidus and substantia nigra via anterograde tracing. Quantitative behavioral phenotyping revealed that Huntington’s disease-induced mice exhibited prolonged survival duration and improved motor deficits in limb coordination and gait symmetry assays. However, since HD is a monogenic neurodegenerative disorder driven by HTT mutations, astrocyte-to-neuron conversion does not address the underlying genetic pathology. Ultimately, both endogenous and directly reprogrammed neuronal populations remain vulnerable to mHTT-mediated proteotoxic degeneration. Therefore, therapeutic intervention through allele-specific genome-editing approaches targeting CAG expansions is imperative to achieve sustained neuronal preservation.

### Amyotrophic lateral sclerosis

ALS, a fatal neurodegenerative disorder, arises from the targeted degeneration of corticospinal and spinal α-motoneurons. This condition manifests through progressive motoneuron depletion, leading to myopathic weakness, paralytic disability, and ultimately fatal respiratory failure. The pathogenic mechanisms driving this neuron-specific degeneration remain unelucidated, with current therapeutic interventions limited to palliative care rather than disease-modifying strategies (You et al., 2023; Xie et al., 2023).

Experimental investigations demonstrate that lineage conversion of human astrocytes into electrophysiologically competent motor neuron-like cells can be achieved with 85% transdifferentiation efficacy through combinatorial modulation using a defined chemical regimen (KFYPR: kenpaullone, fumitremorgin C, Y27632, purmorphamine, retinoic acid). The induced cells exhibited mature neuronal characteristics, including action potential generation and voltage-gated ion channel functionality, covering key hallmarks of spinal α-motoneurons. During transformation, the lineage conversion process exhibited rapid transcriptional dynamics, with NEUROD1 and NGN2 emerging as immediate-early genes upregulated within 24 hours. Induced cells acquired spinal motoneuron-like phenotypes, evidenced by co-expression of canonical markers (HB9/ISL1/CHAT/VAChT) and cholinergic synaptic vesicle packaging capacity. Notably, this transdifferentiation bypassed proliferative neural progenitor states, demonstrating direct astroglial-to-neuron conversion without intermediary expansion phases. The same chemical regimen successfully reprogrammed spinal cord astrocytes in SOD1-G93A ALS murine models into motoneuron-like cells. These lineage-converted cells exhibited compromised viability and increased oxidative damage compared to wild-type motoneurons, thereby recapitulating the pathophysiology associated with ALS. Mechanistically, pathogenic misfolded SOD1 aggregates are established mediators of oxidative stress in astrocytes and motoneurons, primarily through ROS overproduction. Consequently, ALS-derived astrocyte-converted motoneurons exhibited a progressive decline in viability between weeks 2 and 4 post-induction (Zhao et al., 2020). Although the MN-like cells induced in this study are not suitable for cell replacement therapy, they could serve as a valuable cellular model for ALS research and drug screening. Therefore, it is essential to pursue viable therapeutic strategies.

## Challenges, Controversies, and Holistic Considerations in Astrocyte Transdifferentiation

Over the past decade, numerous studies have demonstrated the ability to transform resident glial cells into functional neurons in mice. Most of these studies have achieved this either through the ectopic expression of single or combinatorial fate determinants or by knocking out individual genes. However, many of these findings have not been validated using rigorous lineage tracing methods, leading some researchers to question whether the transformed neurons are indeed derived from astrocytes.

Wang et al. (2021) indicated that NEUROD1 can specifically, rapidly, and efficiently induce mCherry^+^ neurons, but these neurons, which were believed to be transformed from astrocytes, are actually endogenous neurons. First, the immature neuronal marker DCX could not be detected during transdifferentiation following the injection of the NEUROD1 virus, and viral reporter gene-positive (reporter^+^) neurons could not be co-labeled with reactive astrocytes carrying BrdU markers after brain injury. Second, NEUROD1-induced reporter^+^ neurons in normal or injured mouse brains could not be traced using the astrocyte lineage reporter gene YFP. Third, reporter^+^ neurons could still be detected after induction with a mutant, neurologically inactive NEUROD1, suggesting viral mistargeting. This indicates that the hGFAP promoter does not fully target astrocytes and that its targeting can be easily influenced by downstream factors such as NEUROD1. Additionally, the AAV-FLEX system suffers from Cre-independent leaky expression, raising the possibility that endogenous neurons are being mislabeled. Fourth, retrograde labeling reveals that endogenous neurons are the cellular source of NEUROD1-induced reporter^+^ neurons. Finally, while AAV-GFAP-CasRxPtbp1 viruses were ineffective in efficiently down-regulating PTBP1, AAV-hGFAP-mCherry-shPtbp1 succeeded in reducing PTBP1 levels to background levels. However, mCherry+ neurons induced by both methods still could not be traced by the astrocyte lineage reporter gene YFP (Wang et al., 2021). Emerging research now challenges previous reports, indicating that the neuronal-like cells generated by NeuroD1 lack mature neuroelectrical properties, which limits their functional integration into neural circuits (Chen et al., 2025). Effective knockdown of astrocyte PTBP1 using AAV-shRNA or ASO in the substantia nigra and striatum of the hexahydroxydopamine-induced murine model of PD did not result in the conversion of astrocytes into dopamine neurons, and significant AAV leakage was observed (Wang et al., 2021). Similarly, in another study, AAV-shRNA in a murine model of AD also failed to convert astrocytes into neurons (Guo et al., 2022).

In the same year, Rao et al. (2021) demonstrated that NeuroD1 expression did not facilitate the conversion of microglia into neurons, both *in vitro* and *in vivo*; rather, it induced microglial death, even when BCL2 was used for anti-apoptotic rescue. This conclusion contradicts the findings of Brulet et al (2017). Additionally, the study by Rao et al. (2021) showed that significant nonspecific lentiviral leakage into neurons could still be detected after using microglia-specific CX3CR1^+^/CreER mice. Thus, Rao et al. (2021) concluded that the observation by Brulet et al. (2017) regarding NeuroD1-induced conversion of microglia into neurons could be attributed to insufficient microglial culture purity and lentiviral leakage. Subsequently, it was also reported that downregulation of Ptbp1 by CRISPR-CasRx or shRNA was not sufficient to convert Müller glial cells to retinal ganglion cells (RGCs), either in intact mouse retinas or in retinas with almost complete loss of RGCs due to NMDA-induced excitotoxic injury. The conclusion drawn by Xie et al. (2022) that Müller glial cells could be converted to RGCs after Ptbp1 knockdown was proven incorrect by spectral tracing, which showed that leaky AAV mislabeled endogenous RGCs.

One study offers a different perspective on this. Hao et al. (2023) used mGFAP Cre; R26R-YFP transgenic mice and used AAV-CMV-LSL-RFP-Shptbp1 to down-regulate Ptbp1 in astrocytes, detecting many YFP-RFP^+^ NEUN^+^ cells. The study concluded that these YFP-RFP^+^ cells were not the result of non-dependent recombination of viral Cre, as no RFP+ cells were detected when the same virus was injected into the brains of wild-type mice. Hao et al. (2023) proposed the hypothesis that the level of Cre expression in astrocytes varies among different subclasses of astrocytes. When Cre levels were low, the exosomal viral DNA used for RFP expression recombined more efficiently than the chromosomal DNA used for YFP expression, resulting in a large number of YFP-RFP^+^ cells. While Hao et al.’s hypothesis challenges the genetic lineage tracing gold standard, it lacks clear experimental evidence, and further studies are needed to explore it (Hao et al., 2023).

Xiang et al. (2024) performed longitudinal two-photon microscopy to chronologically track the astrocyte-to-neuron transdifferentiation cascade mediated by NeuroD1. Longitudinal monitoring over weeks revealed morphological evolution from astrocytes—characterized by short, tapered processes—to neurons exhibiting elongated neurites and dynamic growth cones. Electrophysiological profiling confirmed that lineage-converted neurons demonstrated synchronous Ca^2+^ oscillations, repetitive action potential firing, and spontaneous postsynaptic currents, with validation through two-photon calcium imaging and whole-cell patch-clamp electrophysiology. These findings indicate functional synaptic integration into endogenous neural networks (Xiang et al., 2024). Liu et al. (2024) engineered AAV9P1, a novel recombinant adeno-associated viral vector incorporating an astrocyte-specific P1 peptide and GFAP promoter that enables dual targeting of reactive gliosis and scar-associated astrocytes. This platform demonstrated enhanced astrocyte specificity with minimal off-target transduction compared with conventional AAV serotypes. In murine traumatic brain injury models, systemic administration of AAV9P1-shPTBP1 via caudal vein induced astrocyte-to-neuron conversion throughout cortical and hippocampal regions, positioning AAV9P1 as a transformative delivery system for *in vivo* neural transdifferentiation research.

A novel investigation exploring the *in situ* conversion of resident astrocytes into functional neurons within an AD murine model demonstrates neuroprotective potential. Four lines of experimental evidence support this mechanism: 1) Immunohistochemical analysis identified transitional cellular populations co-expressing glial and neural markers exclusively in NeuroD1-treated 5xFAD specimens. 2) Comprehensive single-cell RNA sequencing (scRNA-seq) profiling demonstrated a progressive downregulation of astrocytic transcriptional signatures concurrent with an upregulation of neuronal differentiation markers during cellular transdifferentiation. 3) Computational trajectory reconstruction revealed a progressive commitment of NeuroD1-transduced astrocytes toward neuronal lineage specification. 4) Genetic fate-mapping through a Cre-dependent FLEx reporter system confirmed astrocyte-specific activation of the CAG promoter-driven NeuroD1 construct and subsequent neuronal transdifferentiation events. This multimodal approach systematically validates the phenotypic conversion process while maintaining cellular specificity in the context of AD (Wu et al., 2025). These findings provide a strong counter to skeptical voices; however, more evidence is needed before the conclusion of “leakage” can be completely disproved.

## Future Directions for Astrocyte Transdifferentiation Research

Therapeutic conversion of glial cells into functional neurons presents a transformative strategy for addressing cerebrovascular accidents, neural trauma, and degenerative neurological disorders, proposing innovative solutions for central nervous system repair. While experimental validation in murine models and human cellular systems has achieved proof-of-concept status, critical mechanistic complexities surrounding cellular transdifferentiation efficiency and phenotypic stability must be resolved to bridge the preclinical-clinical gap in neuroregenerative interventions.

### Advancing transdifferentiation efficiency

Although the transdifferentiation of astrocytes into functional neurons *in vivo* remains controversial, the inefficiency of this process is a common phenomenon. After cerebral infarction and spinal cord injury, cells produce a large amount of stressful reactive oxygen species. Concurrently, astrocytes face a double blow of oxidative stress during transdifferentiation due to the rapid transformation of metabolic pathways, leading to the excessive accumulation of lipid peroxides within the cells (Yang et al., 2019; González-Nieto et al., 2020). This high level of oxidative stress hinders the smooth transdifferentiation of astrocytes into neurons.

It has been shown that ferroptosis inhibitors, antioxidants, and Bcl-2 can effectively increase the transdifferentiation efficiency of cells while promoting cell survival and neuronal transformation (Gascón et al., 2016). Additionally, early induction of various neuronal mitochondrial antioxidant proteins has been found to improve neuronal transdifferentiation (Russo et al., 2021). Furthermore, electromagnetized gold nanoparticles (AuNPs) and elongated porous gold nanorods (Au Np Rs) can promote the efficient transdifferentiation of mouse fibroblasts into neurons both *in vitro* and *in vivo* when used in combination with transcription factors, significantly enhancing the efficiency of this process. Nanoenzymes also play an important role in ischemic stroke by alleviating ROS overproduction, oxidative brain damage, inflammation, and BBB disruption (Yang et al., 2024). A recent study indicates that conductive microgels enhance oxygen release and electrical stimulation through external alternating magnetic fields, thereby improving neural regeneration during traumatic brain injury reprogramming (Iao et al., 2025). These approaches provide new pathways to improve the efficiency of astrocyte transdifferentiation.

### Selection of suitable astrocytes

Astrocytes play a very important role in CNS tissues while protecting them and maintaining tissue homeostasis after various CNS injuries. Stimulated astrocytes are called reactive astrocytes (Wanner et al., 2013; Sofroniew, 2015). Although reactive astrocytes are also referred to as “glial scarring,” they are not in sync with the definition of scar tissue in other tissues. Reactive astrocytes protect and preserve adjacent functional neural tissue by isolating damaged brain tissue from adjacent surviving neural tissue after damage (Sofroniew, 2015). Loss or attenuation of these cells will result in increased inflammation and spread of serum proteins, increased loss of neural tissue, and detrimental recovery of function (Benner et al., 2013). However, excessive proliferation of reactive astrocytes will impede the extension of neuronal axons and is detrimental to neuronal growth. This dual role of astrocytes - both beneficial and hindering - has been a hot topic of research, especially in the context of glial cell transdifferentiation and regenerative medicine. There is spatial heterogeneity in astrocyte responses within the site of injury. Astrocytes close to the core of the lesion showed high reactivity, proliferation, and extracellular matrix deposition. In contrast, astrocytes in the peri-lesion zone showed reduced reactivity and generated a less inhibitory microenvironment. The difference in reactivity suggests that astrocytes in the peri-injury zone may be more susceptible to transdifferentiation, as their low inflammation and proximity to surviving neurons may facilitate the integration of induced neurons into existing neural circuits, and therefore contribute to the restoration of lost neural function. The immature neurons transformed from astrocytes in the white matter are significantly different from the mature neurons transformed from astrocytes in the gray matter, suggesting an important effect of regional differences on astrocyte transdifferentiation. There is also the obvious limitation that glial cells must be present for transformation to occur. When the damage or degeneration is so severe that a cavity is formed, or when the glial cells themselves are significantly damaged, there is a defect in the glial cell origin that prevents transformation from occurring (Wu et al., 2020). Under such severe injury conditions, it may be necessary to first transplant external cells to fill the cavity for tissue repair. Therefore, it is necessary to identify the appropriate amount of initial cell depletion to maximize the neuroprotective effects of astrocytes and to suppress the potential negative effects of transdifferentiation.

### Bridging the translational chasm

The ongoing divide between experimental and clinical research continues to impede the development of therapies in cellular neuroregeneration. Conventional experimental paradigms using genetically homogeneous juvenile specimens inadequately model the multifactorial pathobiology observed in human age-related cerebrovascular disorders and progressive neurodegeneration. This disparity underscores the imperative to develop pathologically relevant model systems that recapitulate human pathophysiological complexity. Age-associated neurodegeneration manifests multifaceted pathological cascades, including persistent neuroinflammation, reactive astrogliosis, and compromised endogenous repair mechanisms—pathognomonic features systematically underrepresented in conventional juvenile murine systems. Additionally, with the presence of deleterious factors such as reactive oxygen species and inflammatory cytokines in these patients, it remains unclear whether induced neurons degenerate or become damaged over time. Therefore, improving preclinical models to incorporate these age- and disease-related factors, as well as designing clinical trials that carefully account for age-related declines in neuroplasticity, is critical to advancing transdifferentiation therapies.

### Developing advanced targeted delivery and minimally invasive modulation technologies

Most of the current *in vivo* delivery techniques for transcription factors and miRNAs rely on viral vector systems, but such approaches carry significant potential risks. For example, retroviruses or lentiviruses may lead to insertional mutagenesis or even carcinogenesis due to their genomic integration properties, which pose a serious challenge to the safety of clinical applications. In order to balance safety and efficacy, adeno-associated virus (AAV) has become a mainstream vector for gene therapy due to its low immunogenicity. However, its transfection efficiency is insufficient and often requires higher viral doses, and the high concentration may lead to non-targeted tissue infection or off-target effects, so there is an urgent need to explore the dose-effect balance by optimizing the screening of AAV subtypes or the drug delivery strategy (Dai et al., 2024). Compared with viral vectors, chemical small molecule-mediated transdifferentiation technology shows unique advantages: on one hand, it does not require gene integration to avoid the risk of tumorigenesis, and the synthesis process is mature and controllable; on the other hand, existing studies have screened a variety of combinations of compounds that can induce the differentiation of astrocytes to functional interneurons (e.g., GABAergic, glutamatergic subtypes) and enhance the targeting efficiency through novel delivery technologies. However, chemical transdifferentiation still faces multiple challenges: first, the synergistic mechanism of different small molecules is complex, and systematic optimization of the combination scheme is needed to simplify the induction process; second, the lack of BBB penetration may lead to off-target toxicity during systemic administration, and brain-region specific delivery tools are needed to be developed; moreover, how to maintain long-term functional stability of the transformed neurons still needs to be verified in depth. Non-viral delivery systems, including nanoparticles, liposomes and exosomes, offer safer options for delivering transdifferentiation factors. These systems can be engineered to improve stability, control release kinetics, and target specific cell types.

### Ensuring long-term biosafety

**Figure 5:**
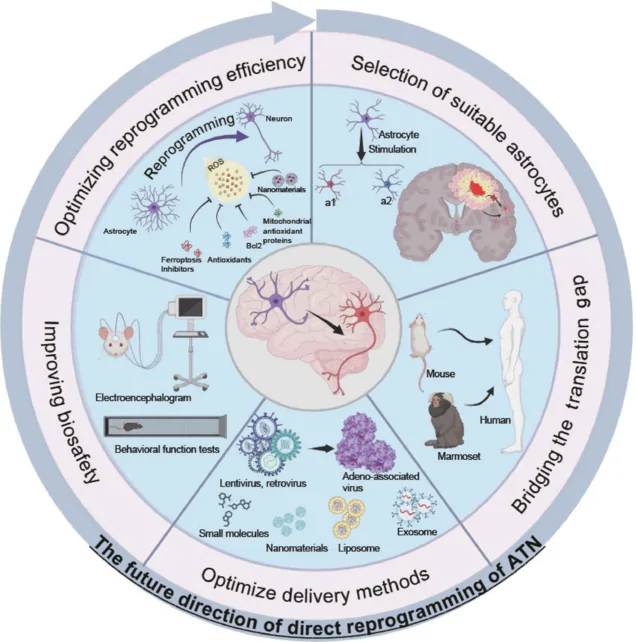
Future direction of transdifferentiation of astrocytes into neurons. Created with BioRender.com. ATN: Astrocyte-to-neuron; ROS: reactive oxygen species.

Some of the transformed cells may lose their original function and fail to become functional neurons, or the induced neurons may fail to integrate into existing circuits, resulting in abnormal electrical activity (e.g., epilepsy). Epileptiform discharges need to be monitored by electroencephalography or optogenetics. Also, induced neurons undergo apoptosis or degeneration *in vivo* and fail to fulfill their original role. Therefore, long-term tracking of the survival rate and function of transformed neurons is needed to optimize the combination of transcription factors or small molecules to improve the efficiency and ensure the quality of transformation. Further, functional impact needs to be assessed in animal models in combination with behavioral tests (such as balance beam and water maze), as axonal misprojection may lead to motor deficits or cognitive abnormalities. Other aspects of safety are as previously described (**Figure 5**).

## Clinical Translation Roadmap and Current Landscape

Significant progress has been made in clinical research within the field of cellular transdifferentiation, spanning applications in neurological injury repair and cancer therapy. Among these efforts, a clinical trial initiated by Stand Up Therapeutics aims to evaluate the safety and preliminary efficacy of the gene therapy STUP-001. This therapy uses an AAV vector to directly transdifferentiate spinal astrocytes into functional neurons, with the goal of improving motor function in patients with chronic spinal cord injury. Another study focuses on malignant glioblastoma and has developed an innovative therapeutic approach based on the oncolytic virus NRG-103. This virus not only specifically lyses tumor cells but also expresses specific transcription factors to transdifferentiate residual glioblastoma cells into non-tumorigenic neurons, thereby suppressing recurrence and activating anti-tumor immunity. Furthermore, in the area of liver disease, a prospective study utilizes clinical-grade human induced hepatocytes (hiHep)—generated via transdifferentiation of human fibroblasts—to construct a bioartificial liver device. This device is used for supportive treatment in patients following major hepatectomy to assess its safety and efficacy in improving liver function and reducing the risk of liver failure (**Additional Table 2**). Collectively, these studies demonstrate the broad therapeutic potential of transdifferentiation technology across diverse diseases.

**Additional Table 2:** Clinical trials related to cell transdifferentiation

ClinicalTrials.gov ID	Starting cell type	Target cell	Disease	Intervention	Vehicle	Study start	Status	FDA-approved drugs
NCT06922890	Astrocyte	Neuron	Spinal cord injury	STUP-001	Adeno-associated viru	2025-07-30	Recruiting	No
NCT06757153	Glioblastoma tumor cells	Non tumor like neuronal cells	Glioblastoma	NRG-103	Not described	2024-12-19	Recruiting	No
NCT05035108	Human fibroblasts	Human-induced hepatocytes	Bioartificial liver after extensive hepatectomy	Not described	Not described	2021-09-05	Recruiting	No

Despite the groundbreaking potential of astrocyte transdifferentiation technology in preclinical studies, its clinical translation remains at an early stage. The delay in clinical application stems from complexities at technical, biological, and regulatory levels. First, the precision and controllability of in situ reprogramming techniques require further optimization. For instance, although AAV vectors are widely used for gene delivery, their targeting efficiency for astrocytes in the human central nervous system may be lower than in rodents, leading to inefficient transdifferentiation or off-target effects. Second, most preclinical studies rely on young, genetically homogeneous mouse models, whereas the varied ages and heterogeneous pathological microenvironments of human patients may significantly affect transdifferentiation outcomes. Moreover, potential risks associated with gene editing tools necessitate long-term follow-up evaluations—existing animal studies, typically with observation periods ≤ 12 months, are insufficient to fully simulate safety concerns over a human lifespan.

## Limitations

This review has several limitations. First, we primarily searched the PubMed, MEDLINE, and Google Scholar databases. While these databases are widely used, they may have overlooked some relevant literature. Second, we included only manuscripts published up to July 2025. Finally, the articles reviewed in this paper were published exclusively in English-language journals.

## Conclusion and Outlook

Neurological disorders such as stroke, spinal cord injury, and neurodegenerative conditions lack curative therapies due to irreversible neuronal loss and impaired regeneration. Astrocyte transdifferentiation-based regenerative strategies represent a paradigm shift by harnessing endogenous cellular resources to directly generate functional neurons. Research demonstrates that combinatorial approaches - using transcription factors, small molecules, or Ptbp1-targeted gene silencing - successfully convert astrocytes into glutamatergic, GABAergic, or dopaminergic neurons both *in vitro* and *in vivo*. These induced neurons significantly restore motor, cognitive, and neural circuit functions in models of cerebral infarction, spinal trauma, and neurodegeneration. Critically, this endogenous transdifferentiation strategy circumvents immune rejection and ethical controversies inherent to exogenous stem cell transplantation while leveraging injury-reactive astrocyte proliferation for intrinsic “self-repair,” substantially enhancing clinical translatability.

This review systematically articulates the tripartite significance of astrocyte transdifferentiation in neural repair. Scientifically, the technology elucidates the molecular mechanisms governing endogenous transdifferentiation, demonstrates targeted plasticity beyond established glial fate restrictions, and establishes new neurodevelopmental paradigms. Practically, its integrated in situ regeneration and functional integration pathway bypasses immunological barriers and ethical dilemmas associated with cell transplantation, while also reducing translational costs. Clinically, it offers scalable solutions for neuronal replacement in refractory conditions such as stroke and PD, advancing patient-specific glia-to-neuron conversion toward clinical implementation.

Distinct from prior syntheses, this work establishes the first multidimensional evolution atlas of astrocyte transdifferentiation, deconstructing transdifferentiation methodologies to reveal core regulatory principles. We innovatively propose a four-dimensional long-term safety assessment framework evaluating neuronal survival, functional integration, tumorigenicity, and epileptogenic risk, addressing previous overemphasis on short-term efficacy. The review further contextualizes these advances within contemporary clinical developments, including the pioneering STUP-001 first-in-human trial.

The scientific implications extend broadly: established transdifferentiation principles successfully generalize to Müller glia and oligodendrocyte precursor cells, covering > 90% of CNS pathological contexts. Clinically, the strategy provides cross-disease regenerative solutions leveraging shared mechanisms between neurodegeneration and acute injury.

Despite persistent challenges, this technology opens new therapeutic dimensions. Future integration of single-cell multi-omics, organoid modeling, and AI-driven drug screening promises to achieve on-demand neuronal subtype specification, accelerating personalized regenerative medicine toward clinical reality. These advances hold potential to fundamentally reshape neurorestorative frameworks and offer functional recovery for millions with neurological disorders.

## Additional files:

***Additional Table 1:*** *Representative transcription factors involved in in vitro and in vivo direct astrocyte-to-neuron conversion and transdifferentiation studies.*

***Additional Table 2:*** *Clinical trials related to cell transdifferentiation.*

## Data Availability

*All relevant data are within the paper and its Additional files.*
